# Population dynamics of Pinfish in the eastern Gulf of Mexico (1998-2016)

**DOI:** 10.1371/journal.pone.0221131

**Published:** 2019-08-22

**Authors:** Meaghan E. Faletti, Dinorah H. Chacin, Jonathan A. Peake, Timothy C. MacDonald, Christopher D. Stallings

**Affiliations:** 1 College of Marine Science, University of South Florida, St. Petersburg, FL, United States of America; 2 Fish and Wildlife Research Institute, Florida Fish and Wildlife Conservation Commission, St. Petersburg, FL, United States of America; Universidad del Valle, COLOMBIA

## Abstract

Forage fishes play an important role in marine ecosystems by transferring energy and nutrients through the food web. The population dynamics of forage species can therefore have cascading effects across multiple trophic levels. Here, we analyzed a 19-year dataset on Pinfish (*Lagodon rhomboides*) across four eastern Gulf of Mexico estuaries to investigate population dynamics, inter- and intra-annual synchrony, metapopulation portfolio effects, growth, and habitat effects. Young-of-year growth rates did not differ among estuaries. The population dynamics of these four systems were stable in the long-term, but highly dynamic inter-annually. Intra-annual dynamics were stable and predictable despite variation in long-term means. Some estuaries exhibited positive inter-annual synchrony, and all four estuaries were synchronous intra-annually. There was evidence for stronger portfolio effects for the entire four-estuary metapopulation, as well as for the two northern estuaries while the southern estuaries appeared to act as a single population. Submerged aquatic vegetation was by far the most important predictor for both presence and abundance of Pinfish. It is important to understand the factors driving forage fish population fluctuations to better predict ecosystem effects, including those to species of economic and ecological importance. These predictors can be useful for the implementation of ecosystem-based management decisions.

## Introduction

‘Forage fishes’ are defined as small- to intermediate-sized schooling fishes that serve as prey for numerous marine predators [1]. These species fill crucial intermediate trophic levels through schooling behaviors, fast growth, and high abundances that make them a common target as prey [2]. They also fill fundamental niches in energy and nutrient transfer through marine food webs due to their planktivorous and herbivorous diets, relatively high lipid content [3,4], and role as a major prey item in the diets of large fish, seabirds, and marine mammals [5]. In addition to their ecological roles, forage fishes contribute $16.9 billion annually to global fisheries through direct harvest for fish oil and consumption, and indirect support of other fisheries [1]. High fishing intensity can potentially lead to forage fish population collapse [6, 7] and cause pronounced changes in ecosystem structure and function [8, 9]. Conversely, high fishing intensity on predatory fishes can lead to trophic cascades, increases in forage fish abundance, and decreases in biodiversity throughout an ecosystem [10, 11, 12]. Studying the population dynamics of low trophic level species is important for understanding energy transfer and food availability for higher trophic levels. Changes in demographics or population structure of prey species can influence those of their predators, leading to effects throughout a community [2, 13]. These bottom-up effects have been well-studied in many systems and are known to act in conjunction with top-down effects on community structure [13, 14]. Bottom-up effects can vary greatly across scales, thus it is important to understand the dynamics of various trophic levels on multiple spatial and temporal scales [12, 15]. The vulnerability and importance of forage fishes highlight the need for conservation efforts focused on these populations and implementation of ecosystem-based management.

Pinfish (*Lagodon rhomboides*) are an abundant and ubiquitous marine forage fish in eastern Gulf of Mexico (eGOM) estuaries. Pinfish meet several of the required characteristics to be considered a ‘forage fish’ by the standards set forth by Pikitch et al. [1], as the species is a primary consumer, maintains an intermediary trophic position throughout its life, and is a major conduit of nutrients to higher trophic levels [16]. Due to its role within eGOM ecosystems, it has been classified within the forage fish functional group in Atlantis models used for food web modeling [17]. In addition to its ecological importance, a commercial fishery exists for Pinfish in Florida, mainly for use as bait in other commercial and recreational fisheries [18]. Despite its recreational and commercial contributions, and its role as an important prey item, a formal stock assessment has yet to be completed for this species [19].

Juvenile Pinfish settle in Florida seagrass beds from November to March [20]. Young-of-year (YOY) Pinfish then remain and grow in seagrass beds, exhibiting an ontogenetic diet shift from a planktivorous diet to an herbivorous one in which they forage on epiphytic algae [21, 22, 23, 24]. Following maturation (~110mm) [25], adult Pinfish migrate offshore to spawn between October-May [26, 27, 28, 29], although some individuals may remain inshore year-round [22]. Due to the use of both inshore and offshore habitats during different life stages and its high abundance [30], Pinfish serve as prey to a wide range of predators both inshore and offshore and serve as a nutrient subsidy to offshore food webs [16]. Predators include economically-important fisheries species such as Spotted Seatrout, *Cynoscion nebulosus* [23, 31, 32, 33], Red Drum, *Sciaenops ocellatus* [34, 35], and Gag, *Mycteroperca microlepis* [17, 36, 37], as well as seabirds [38, 39, 40, 41], and marine mammals [40].

Given the critical ecological roles Pinfish play in eGOM trophic dynamics, it is important to understand the spatiotemporal patterns in Pinfish population dynamics and the factors potentially driving fluctuations. In this study, a long-term dataset of Pinfish density and biomass was analyzed across four major estuaries in the eGOM. We also apply the theory of portfolio effects to a Pinfish metapopulation to assess the long-term stability of the population when the individuals of a population are “invested” in multiple geographically separate estuaries across the eGOM. The principles of portfolio theory from the field of economics (*sensu* Markowitz [42]) have been applied to a handful of biodiversity studies to assess the security of natural diversity [43] through the calculation of return–risk ratios of ecosystem “portfolios.” Here, we use this technique to assess the long-term security of a population of Pinfish in the eGOM. Knowledge on portfolio effects and long-term population stability can help inform management on the vulnerability of fish stocks to perturbations.

The objectives of this study were to: 1) analyze the long-term, inter- and intra-annual population dynamics of Pinfish across these estuaries to describe the spatial and regional patterns in long-term mean and variation, 2) investigate the inter- and intra-annual population synchrony among these four estuaries, and measure the strength of metapopulation portfolio effects, 3) analyze regional variation in YOY size and growth, and determine if this variation influenced the density-biomass dynamics within each estuary, and 4) model the habitat variables that were related to long-term Pinfish population dynamics.

## Methods

### Sampling design

**Fig 1:**
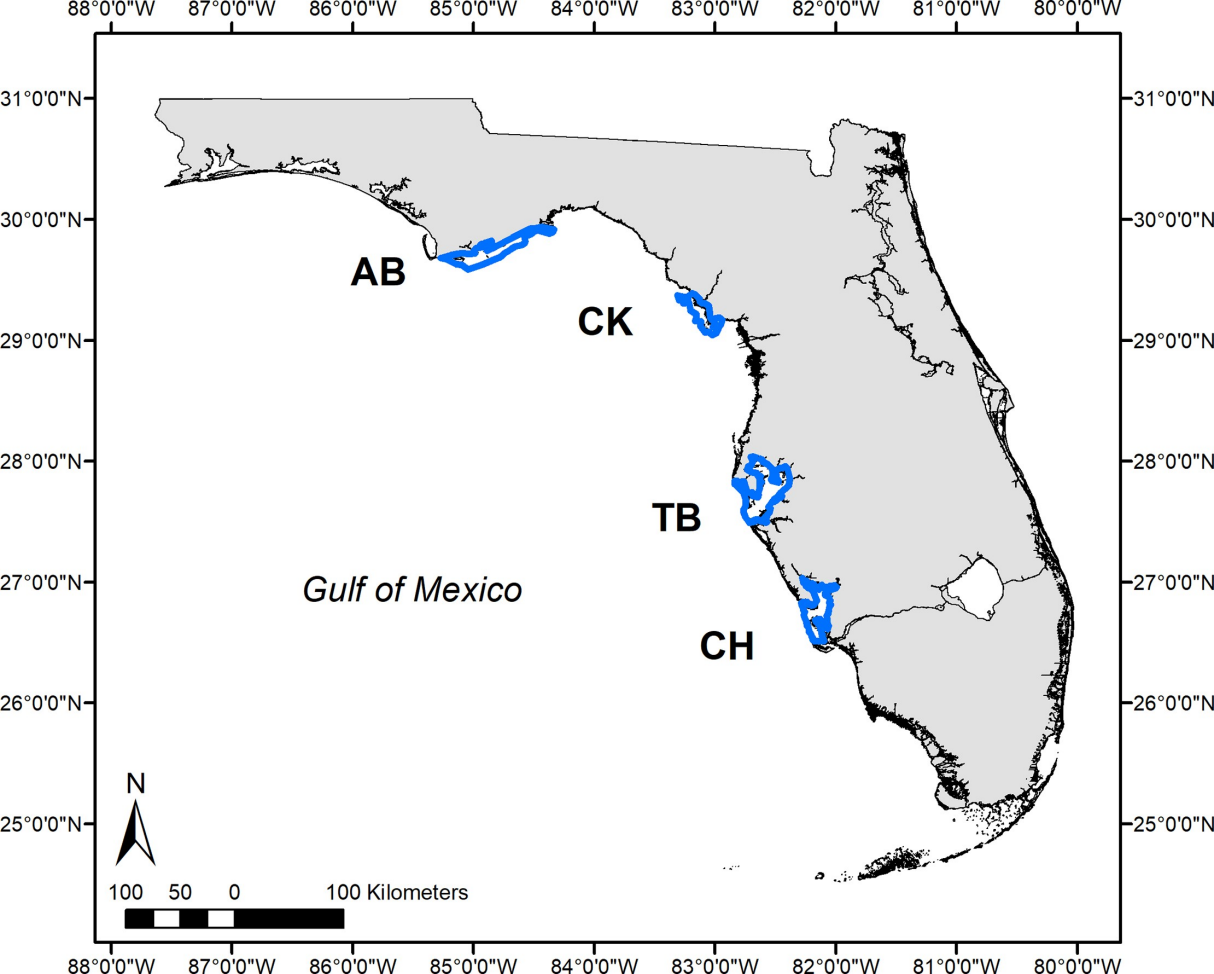
Map of study sites. Blue polygons represent areas sampled by FWRI’s FIM program from 1998–2016 in four eastern Gulf of Mexico (eGOM) estuaries: Apalachicola Bay (AB), Cedar Key (CK), Tampa Bay (TB), and Charlotte Harbor (CH).

Specimen collections were conducted via standard protocols by the Florida Fish and Wildlife Research Institute (FWRI) Fisheries Independent Monitoring (FIM) program. These protocols were authorized by the Florida Fish and Wildlife Conservation Commission for state fisheries and conservation research. All animals counted and measured for this dataset were released alive in the field. Stratified random sampling was conducted monthly from 1998–2016 by the FWRI FIM program in each of four estuaries: Apalachicola Bay (AB), Cedar Key (CK), Tampa Bay (TB), and Charlotte Harbor (CH; Fig 1). Strata were defined by spatial zones (based on geographic, hydrologic, and logistical criteria), water depths, and habitat types (submerged aquatic vegetation [SAV], unvegetated, and shoreline). Each estuary was subdivided into 1 nautical mile^2^ (nmi^2^) grids and stratified by depth. Sampling was conducted using a 21.3 m center-bag seine with 3.2 mm mesh netting that is restricted to use in depths <1.8m. Therefore, grids with a minimum depth between 0.1 and 1.8 m were randomly selected within each estuary for sampling. Within each randomly selected grid, a 183 m^2^ microgrid was randomly selected as the starting point to search for the designated habitat [44]. At each sampling site, environmental variables including salinity and water temperature (°C) were measured with a YSI handheld multiparameter water quality meter, and the percent cover of SAV was visually estimated in ten percent increments. A seine haul consisted of pulling the seine 9.1 m with a 15.5 m line maintaining a consistent net opening, resulting in a sampled area of 140 m^2^. After net deployment, the sample was retrieved by pulling the leads around a pole to concentrate the sample in the bag. All Pinfish were counted and a subsample of up to ten randomly selected individuals was measured for standard length (SL) in millimeters (mm) before being released alive. Mean number of seine hauls per month varied between 9–20 per estuary (See S1 Table).

### Analysis

To examine Pinfish population dynamics, catch abundances were converted to densities by dividing the number of Pinfish collected in each sampling event by the total area sampled with the gear. Seine hauls conducted a minimum of 5m away from the shoreline were used in these analyses. To analyze spatial patterns among estuaries, density (fish/100m^2^) for each net haul was calculated and then averaged over all months and years for each estuary. Second, mean density was calculated over all months in each estuary per year to examine inter-annual patterns. Third, density was averaged over years in each estuary per month to explore intra-annual patterns. Mean biomass (g/100m^2^) was estimated to compare dynamics between Pinfish density and biomass. Lengths of the measured subsample of Pinfish were extrapolated to the remaining unmeasured Pinfish sample before biomass was calculated using the length-weight relationship:

$$W=a\times SL^{b},$$ (1)

where W is weight in grams, SL is standard length in millimeters, and a and b are length-weight constants. The regression parameters (a and b) were derived from estuary-specific length and weight data from subsamples of Pinfish caught during monthly inshore fisheries-independent surveys (2001–2017; n = 3,517).

To test for population synchrony, time-series analyses were conducted on the Pinfish density data. To investigate inter-annual synchrony, the density data were detrended to remove seasonal signals. For intra-annual synchrony, analyses were focused only on the seasonal signals. Then cross-correlations were conducted for each estuary pair to test for inter- and intra-annual synchrony.

The principles of portfolio theory from the field of economics (*sensu* Markowitz [42]) have been applied to a handful of biodiversity studies to assess the security of natural diversity [43] through the calculation of return–risk ratios of ecosystem “portfolios.” Here, this technique is applied to a Pinfish metapopulation to assess the long-term stability of the population when the individuals of a population are “invested” in geographically separate estuaries. Metapopulation stability is a direct function of local and large-scale abundance and population synchrony [45, 46], while extirpation risk is heightened by low population variability, decreasing abundance and synchronous population dynamics [47]. For each portfolio, we calculated the average coefficient of variation portfolio effect (CVPE) [48]:

$$CVPE=\frac{\left(CV_{p1}+CV_{p2}+\cdots +CV_{pn}\right)/n}{CV_{m}},$$ (2)

where *CV*_*p1…n*_, are the coefficients of variation for each local population, *n* is the number of local populations, and *CV*_*m*_ is the coefficient of variation for the overall metapopulation. The CVPE quantitatively describes the mean variation of local populations with variance dampening from the metapopulation. Larger values represent stronger portfolio effects, or greater variance dampening, while low values represent no variance dampening or complete synchrony [47]. A CVPE = 1.0 therefore effectively represents a single homogeneous population while CVPE >1 represents a population with asynchronous dynamics that is more stable than a single homogeneous population.

A month-to-month comparison of mean SLs was conducted to estimate Pinfish growth rates. A bimodal distribution in standard length was observed in December, indicating presence of a second, smaller cohort within each estuary. This was confirmed through the calculation of bimodality coefficients (AB: 0.79; CK: 0.74; TB: 0.61; CH: 0.67), where a value >0.55 suggests bimodality [49]. Due to the potential for the bimodality coefficient to incorrectly assume a bimodal distribution from a highly skewed unimodal distribution [50], we also conducted the Hartigan’s dip test [51], which confirmed significant multimodality in each estuary (all p < 2e-16). This smaller cohort was split into a “month 0” to represent the recruitment of smaller fish for the beginning of the following year. Instantaneous growth coefficients were additionally calculated for Pinfish annually. The assessment of growth coefficients in each estuary assumed limited migration of Pinfish among estuaries, however, other factors could affect size class structure such as size selective mortality and egress. Thus, growth rates were approximated with mean SL data with an emphasis on months April through July to reduce biases related to settlement (January-March) and ontogenetic movements of juveniles (August-December). Following the approach of Nelson [20], growth was estimated with the model:

$$\ln\left(L_{t}\right)=\ln\left(L_{0}\right)+G\times t,$$ (3)

where G is the instantaneous growth rate (per month), L_t_ is the monthly mean SL (mm), L_0_ the theoretical SL at which Pinfish recruit to each estuary, and t is time in months.

A zero-altered negative binomial (ZANB) analysis was used to explore which environmental variables were related to the observed variation in Pinfish presence and density for each individual estuary. A ZANB model was selected due to overdispersion of both zeroes and non-zero data. Four methods were tested (zero-inflated negative binomial, zero-inflated Poisson, and zero-altered Poisson). Akaike Information Criteria (AIC) model selection was used to determine the most appropriate model. The ZANB approach was the most appropriate, with higher R^2^ and lower AIC than the other three models. This analysis consists of two generalized linear models: a binomial distribution was used to model presence-absence probability, and a negative binomial distribution was used to model density patterns. Explanatory variables included percent cover of SAV, water temperature, salinity, water depth, SAV^2^ (to account for non-linear saturation effects of habitat structure), and SAV-temperature interaction term. A stepwise model selection was conducted via a combination of forward addition and backward elimination on both the presence-absence and abundance models for each estuary based on minimization of the AIC. To test for multicollinearity among model predictors, a tolerance test was conducted on the full model excluding interaction and power terms for each of the presence-absence and abundance models by estuary. At a tolerance level of 0.5, there was no evidence of multicollinearity among any of the predictors in both the presence-absence and density models for all estuaries (VIF range: 1.001–1.352), which gave sufficient justification to include all predictors in the selection process. Analyses were conducted using the *MASS* and *car* packages for R Statistical Computing Environment [52, 53, 54].

## Results

**Fig 2:**
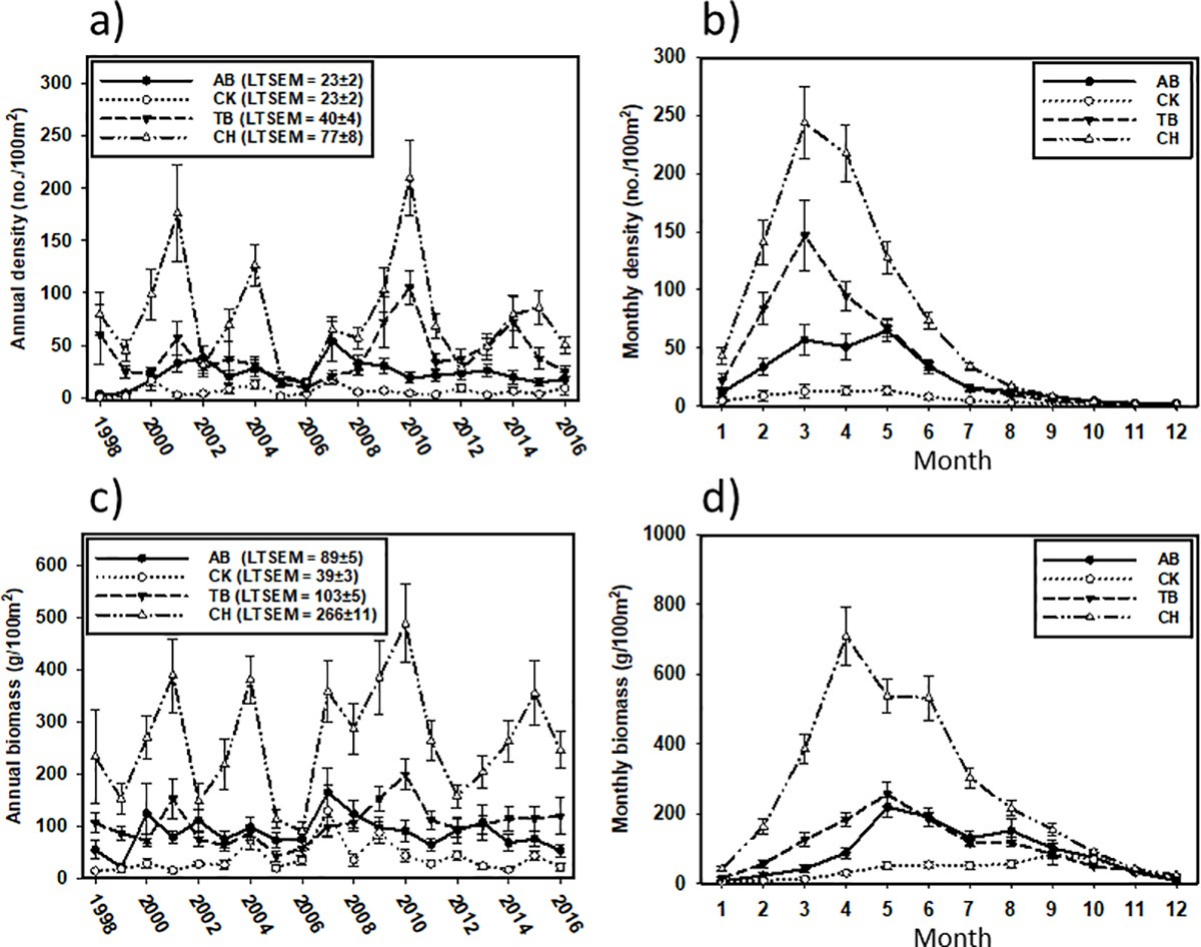
Inter- and intra-annual Pinfish densities and biomass. Inter- (left column) and intra-annual (right column) population dynamics of Pinfish density (top row) and biomass (bottom row) from 1998–2016. Long-term means (± SE) are reported for each estuary in the legends of the inter-annual plots. The four plots display: a) inter-annual density, b) intra-annual density, c) inter-annual biomass, d) intra-annual biomass.

Patterns in mean density and biomass were similar among estuaries but both varied by as much as an order of magnitude between estuaries (Fig 2A–2D). Both interannual mean density (Fig 2A) and biomass (± standard error; Fig 2C) were highest in CH (76.80 ± 8.40 no./100m^2^; 266.18 ± 11.46 g/100m^2^), followed by TB (40.43 ± 4.42 no./100m^2^; 103.18 ± 4.79 g/100m^2^), AB (23.17 ± 2.39 no./100m^2^; 89.19 ± 5.48 g/100m^2^), then CK (6.37 ± 0.93 no./100m^2^; 38.64 ± 3.45 g/100m^2^). Intra-annual densities of Pinfish increased across the four estuaries during the early months of the year and peaked in March-May, for all years combined (Fig 2B). The southern estuaries tended to have a one-month peak in density while the northern estuaries had more prolonged peaks lasting three months. Intra-annual biomass increased across all estuaries from January through April-May for AB, TB, and CH, and through September for CK and subsequently declined through December (Fig 2D).

### Population synchrony

There was positive inter-annual synchrony (with a lag between 0–3 months) in density between the two northern estuaries (AB and CK, r = 0.30) as well as the two southern ones (TB and CH, r = 0.53), and negative synchrony between the two middle estuaries (TB and CK, r = -0.18; all other estuaries were asynchronous at the inter-annual scale) (Table 1A). For biomass, there was positive inter-annual synchrony between all pairs (r = 0.14–0.43), except TB and AB, which were asynchronous (Table 1B).

**Table 1:** Inter- and intra-annual population synchrony between estuaries. Cross correlation coefficients (*r*) of peak synchrony measured within 0–3 month lags between estuaries for a) inter-annual density, b) inter-annual biomass, c) intra-annual density, and d) intra-annual biomass. Significant correlations are in bold and the peak lag time in months is reported in parentheses.

**a) Density inter-annual**				**b) Biomass inter-annual**			
	**AP**	**CK**	**TB**		**AP**	**CK**	**TB**
**AP**				**AP**			
**CK**	**0.30 (2)**			**CK**	**0.37 (1)**		
**TB**	0.10 (3)	**-0.18 (0)**		**TB**	0.11 (1)	**0.14 (3)**	
**CH**	0.10 (2)	0.08 (1)	**0.53 (1)**	**CH**	**0.22 (2)**	**0.21 (0)**	**0.43 (1)**
**c) Density intra-annual**				**d) Biomass intra-annual**			
	**AP**	**CK**	**TB**		**AP**	**CK**	**TB**
**AP**				**AP**			
**CK**	**0.99 (0)**			**CK**	**0.77 (1)**		
**TB**	**0.88 (1)**	**0.91 (1)**		**TB**	**0.91 (1)**	**-0.85 (3)**	
**CH**	**0.89 (1)**	**0.92 (0)**	**0.99 (0)**	**CH**	**0.92 (1)**	**-0.92 (3)**	**0.92 (0)**

**Fig 3:**
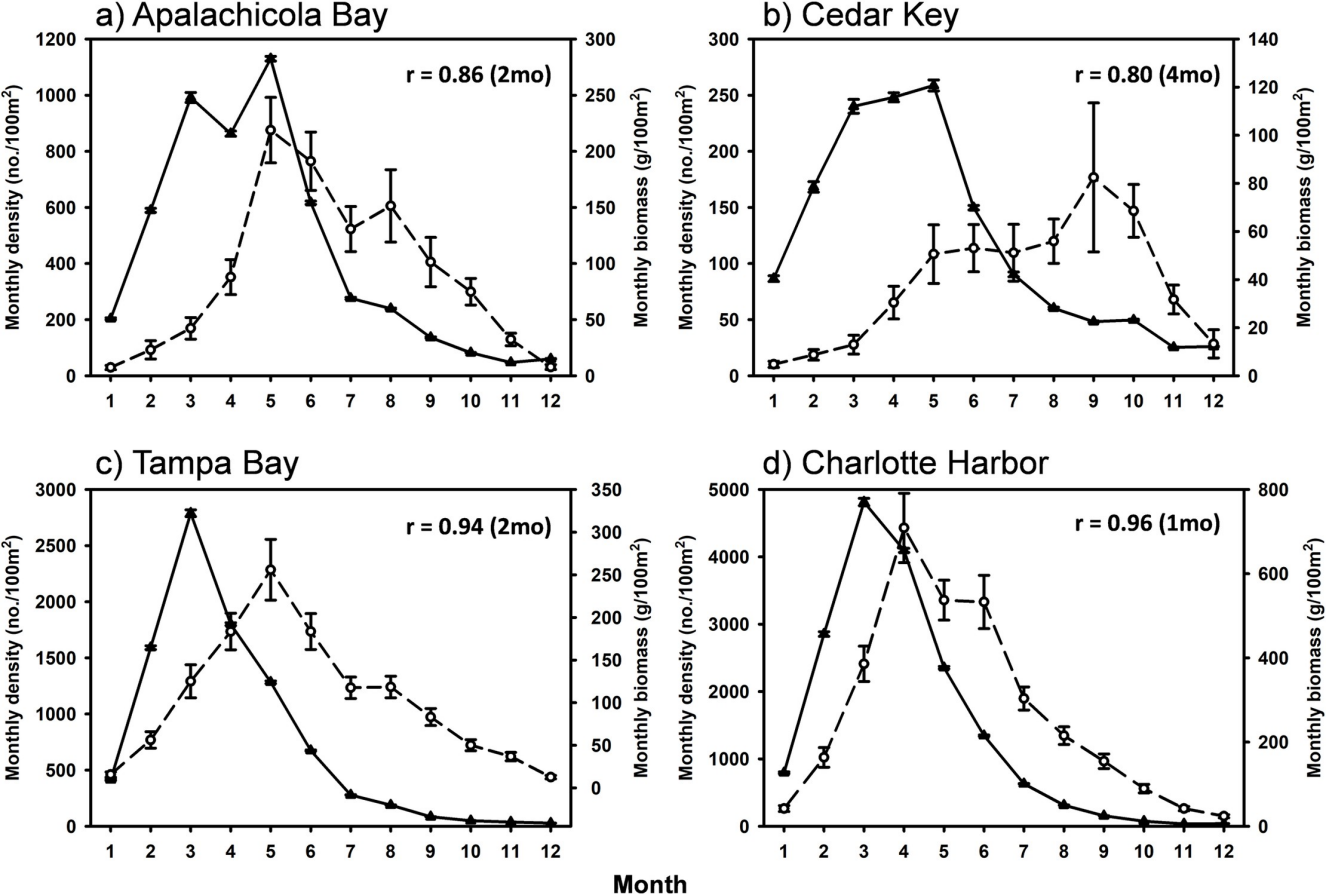
Mean monthly Pinfish dynamics. Mean monthly density (black triangle) and biomass (open circles) of Pinfish from 1998–2016. Months are in order of calendar year (i.e., 1 = January, 12 = December). Cross correlation coefficients and lags reflect peak synchrony between density and biomass within each estuary.

All four estuaries were positively synchronous with each other in intra-annual density (r = 0.88–0.99; Table 1C), although the two northern estuaries (AB and CK) generally lagged one month behind the two southern estuaries (TB and CH). Likewise, biomass was generally positively synchronous intra-annually among estuaries (r = 0.77–0.92), however CK was negatively synchronous with both TB (r = -0.85) and CH (r = -0.92; Table 1D). Within each estuary, peak positive synchrony between density and biomass lagged by one month in CH (r = 0. 0.96; Fig 3D), two months in AB (r = 0.86; Fig 3A) and TB (r = 0.94; Fig 3C), and four months in CK (r = 0.80; Fig 3B).

### Metapopulation portfolio effects

**Fig 4:**
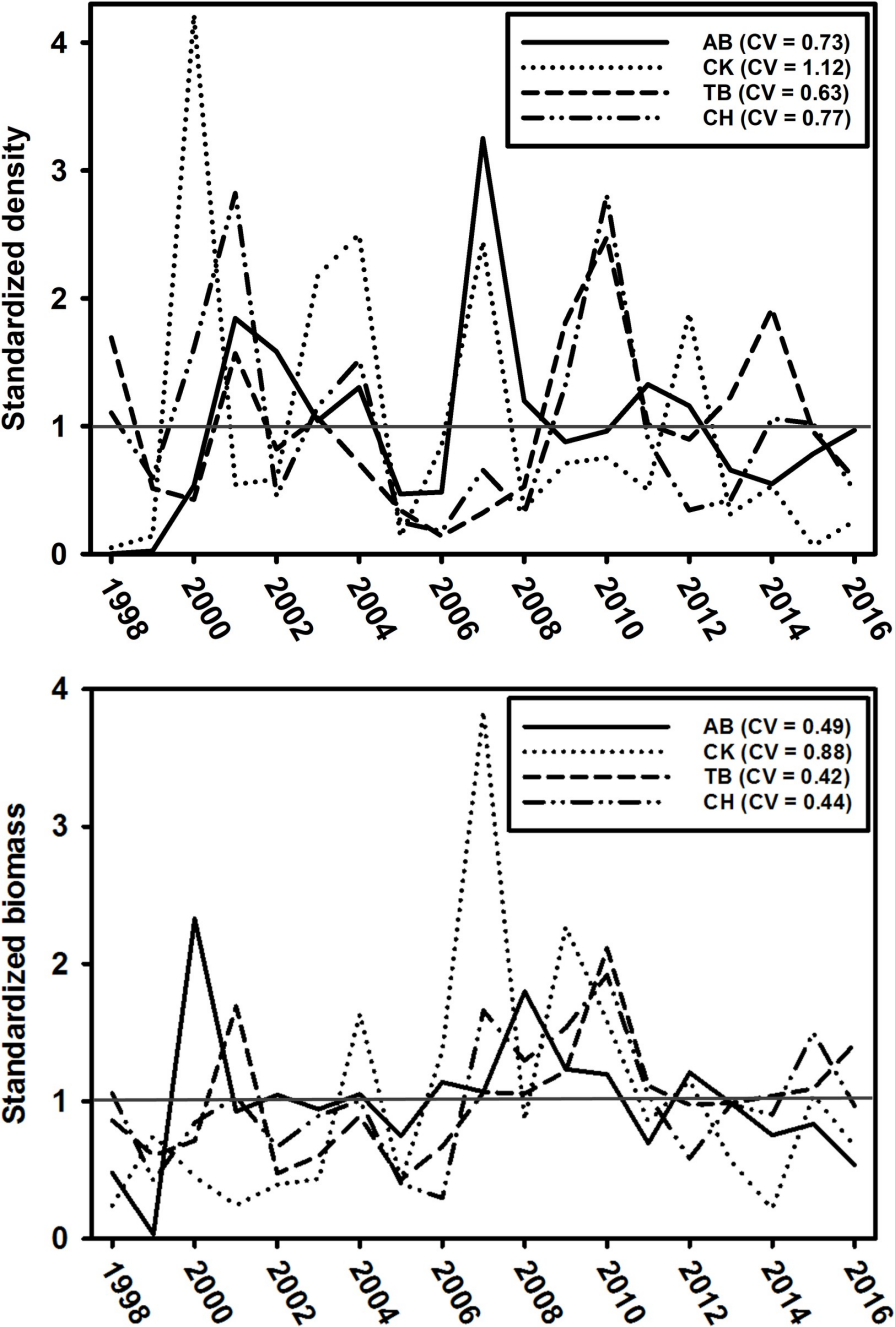
Standardized density and biomass. Standardized density (top panel) and biomass (bottom panel), expressed as the ratio of annual to long-term mean values (horizontal dark gray line). Coefficient of variation (CV) values are provided for each metric in each estuary.

**Fig 5:**
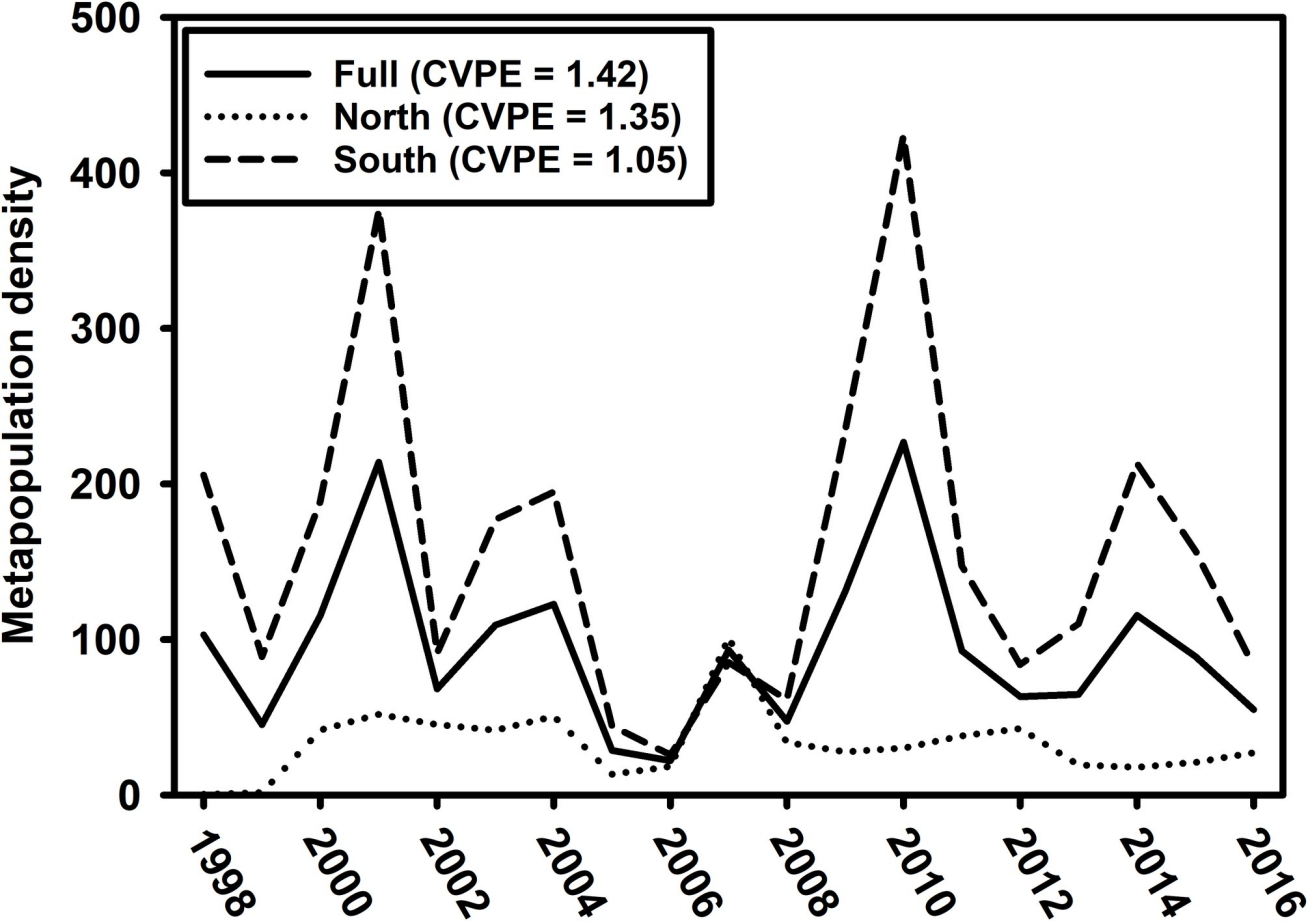
Metapopulation portfolio effects. Long-term mean metapopulation density across all four estuaries (full), two northern estuaries (AB and CK combined), and two southern estuaries (TB and CH combined). The coefficient of variation portfolio effect (CVPE) values are reported for each metapopulation.

The dynamics in peak inter-annual density (three-month moving average) were relatively similar in AB (CV = 0.73), TB (CV = 0.63), and CH (CV = 0.77). Density dynamics were more variable in CK (CV = 1.12). Qualitatively, the peak inter-annual biomass dynamics reflected those for density but were less variable (Fig 4). Specifically, they were similar in AB (CV = 0.49), TB (CV = 0.42), and CH (CV = 0.44), but more variable in CK (CV = 0.88). The magnitude of portfolio effects varied among metapopulations. The full, four-estuary portfolio effect was 42% more stable than a single homogenous population (CVPE = 1.42; Fig 5). The portfolio effects differed between the northern (CVPE = 1.35) and southern (CVPE = 1.05) metapopulations (Fig 5).

### Size and growth

**Fig 6:**
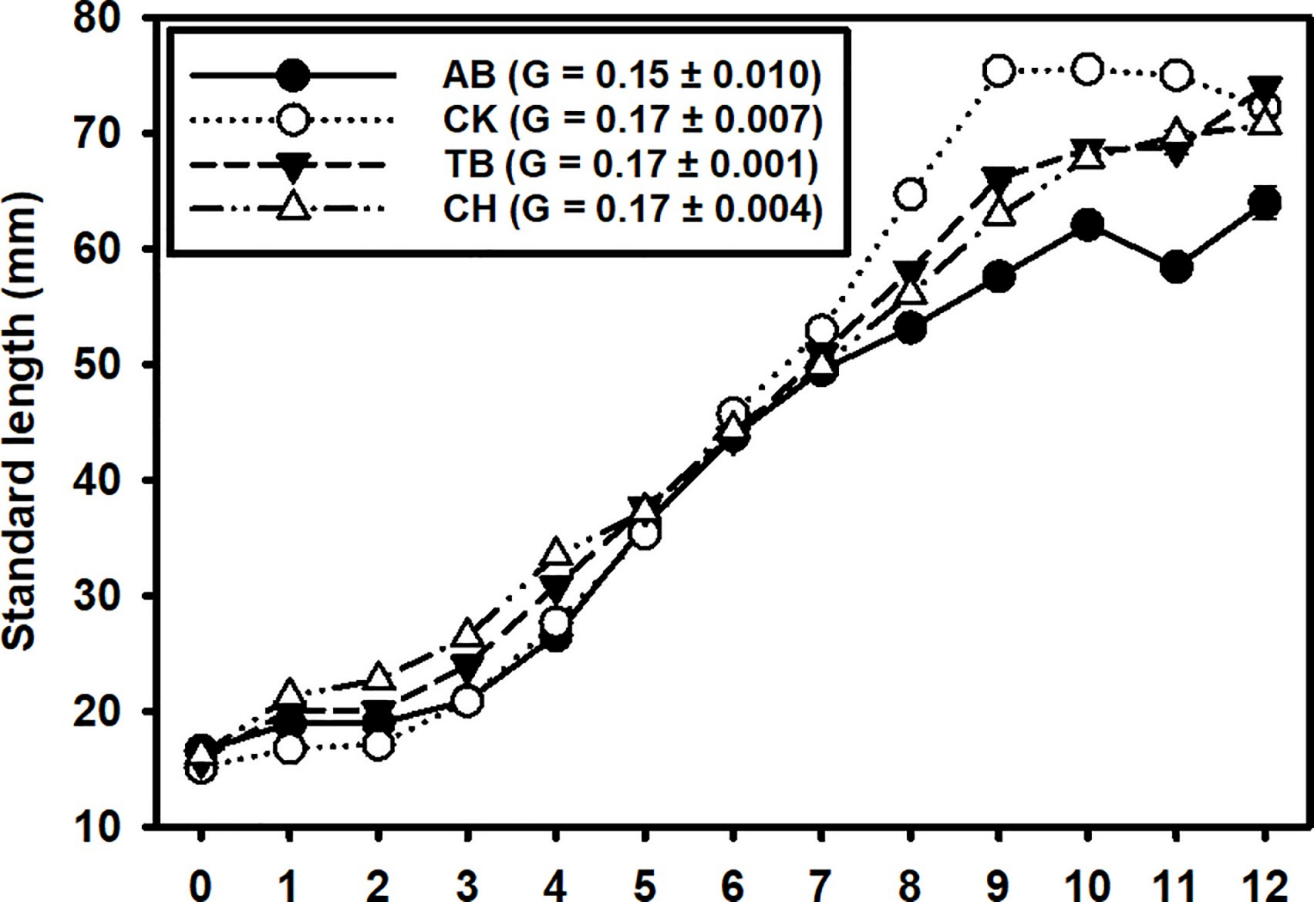
Pinfish growth. Spatio-temporal variation of mean Pinfish standard length among estuaries intra-annually. Means (± SE) of the instantaneous growth rates (G) between April and July for each estuary are reported in the legend. Months are in order of calendar year (i.e., 1 = January, 12 = December), with month 0 representing sizes from the smaller cohort of new YOY recruits in December of the previous year.

Pinfish SL increased in all four estuaries between December, when Pinfish began to recruit, and October of the following year (mean increase of 45–55 mm SL; Fig 6). Mean SLs were smallest in December and January, increased during the summer and early fall, and generally reached an asymptote by October. Mean SLs were similar among the four estuaries through July after which Pinfish were noticeably larger in CK, smaller in AB, and of intermediate size in TB and CH through the remainder of the calendar year. Instantaneous growth rates (G) were similar across estuaries (0.15 to 0.17).

### Habitat variables

Among the habitat variables included in the ZANB model, SAV explained the most variability in both Pinfish presence (Table 2A) and density (Table 2B) in all four estuaries. Submerged aquatic vegetation explained 2–38 times more variability than each of the other significant terms for presence and 2–11 times more variability for density. SAV was followed by the quadratic function of SAV (SAV^2^), which explained more variability than the remaining significant terms (Salinity, Temperature, Depth, SAV*Temperature) for both presence and density of Pinfish.

**Table 2:** Zero-Altered Negative Binomial (ZANB) model outputs. The ZANB analysis consists of two generalized linear models: a zero adjusted (binary) model for presence-absence probability (a), and a negative binomial (count) model for density (b). Values reported are standardized coefficients.

**a) Presence**						
**Estuary**	**SAV**	**Salinity**	**Temperature**	**Depth**	**SAV**^**2**^	SAV*Temperature
**AB**	2.8822***	NS	0.3705***	**0.0765**	-1.6454***	0.1501**
**CK**	0.9183***	NS	NS	**0.0698**	NS	NS
**TB**	2.0577***	0.6921***	NS	NS	-0.7783***	NS
**CH**	1.7210***	0.6606***	0.1891***	0.1863***	-0.4483*	0.1605***
**b) Density**						
**Estuary**	**SAV**	**Salinity**	**Temperature**	**Depth**	**SAV**^**2**^	SAV*Temperature
**AB**	0.9330***	-0.0851*	-0.3825***	0.1304***	NS	0.1422***
**CK**	1.8860***	-0.3579***	-0.3354***	NS	-0.8332***	NS
**TB**	1.0671***	0.3858***	-0.6767***	-0.1017**	NS	NS
**CH**	0.8109***	0.4445***	-0.5587***	0.1460***	-0.313*	NS

Significance:

* = p < 0.05

** = p < 0.01

*** = p < 0.001

NS = Not Selected

The four remaining terms in the ZANB model (in descending order of importance based on the standardized coefficients: salinity, temperature, SAV*temperature, and depth) were positively associated with presence (binary model, Table 2A). Salinity explained Pinfish presence in the two southern estuaries (TB and CH), but not the two northern estuaries. Temperature and the SAV*temperature interaction term accounted for significant variability in the presence of Pinfish in the southernmost (CH) and northernmost (AB) estuaries (Fig 2B).

There was a significant, negative relationship between temperature and Pinfish density in all four estuaries. Salinity also had a significant relationship with density in all four estuaries but with different directionality: positive in the southern (TB and CH) and negative in the northern (AB and CK) estuaries. There was a significant, negative relationship between temperature and Pinfish density for all four estuaries, but SAV*Temperature only accounted for a significant amount of variation in AB. The effects of depth were significant in every estuary except CK, although the magnitude of the relationships was relatively small compared to the other variables.

## Discussion

In this study we analyzed nineteen years of Pinfish catch data from four major estuaries in the eGOM to investigate the population dynamics of one of the most abundant forage fishes in the region. The results add to our understanding of the spatial, inter-annual, and intra-annual patterns of density and biomass for this ecologically important species. Our results showed that although Pinfish population dynamics varied spatially and temporally, there were similar inter- and intra-annual patterns of density and biomass among the four estuaries. Furthermore, we found that both presence and density of Pinfish were related primarily with SAV coverage, and to a lesser degree with other environmental factors including temperature, salinity, and depth.

Density and biomass varied greatly across the four estuaries, possibly due to variation in the quality of suitable juvenile habitat (i.e., seagrass coverage, suitable temperature and salinity ranges) or differences in larval supply. Southern estuaries (CH and TB) had far greater density and biomass than northern estuaries (CK and AB). Mean density and biomass with all years combined were two times higher in CH than TB, with even greater differences between CH and the two northern estuaries (AB and CK). The differences in both density and biomass across estuaries could result from estuarine-specific variation in environmental variables. For example, seagrass beds at higher latitudes experience larger seasonal fluctuations in temperature and solar radiation, which can lead to leaf necrosis and lower seagrass biomass [55], potentially reducing their ability to support Pinfish. In addition, regional variation in dominant seagrass has been observed, with higher cover of turtle grass (*Thalassia testudinum*) in the south and manatee grass (*Syringodium filiforme*) in the north [30]. The wide, flat blades of turtle grass may support more epiphytic algae, which is a key food source for Pinfish [21, 22, 56], compared to relatively thinner and cylindrical structures of other seagrass species [57].

Variation in Pinfish density between northern and southern estuaries could also be driven by differences in larval supply. Pinfish are assumed to spawn offshore [26, 40] along the West Florida Shelf (WFS) and successful settlement occurs when larvae are transported to nearshore estuaries. This transport can be explained by modeled eGOM hydrodynamic patterns, particularly the timing of offshore water delivery across the WFS. Interactions between the Gulf of Mexico Loop Current and the shelf slope affect the magnitude and direction of offshore water moving inshore [58] and could therefore influence larval transport and recruitment of Pinfish to eGOM estuaries. In years when nearshore waters of the TB and CH regions—and AB to a similar but lesser degree—are renewed during protracted upwelling circulation, conditions are not favorable for transport to the CK region [59], and vice-versa. These processes could influence the timing of recruitment pulses to these estuaries along the WFS, leading to the observed inter-annual trends [60, 61]. The CK estuary (i.e., the greater Big Bend region) is located inshore of a very wide portion of the shelf, so larvae may have longer distances to travel to reach this estuary compared to the other estuaries.

Although Pinfish density and biomass were highly dynamic in the region, the populations were stable across these four systems in the long-term, despite the occurrences of severe stressors such as severe cold events [62], red tide events [63], and seagrass die-offs [64]. This study revealed evidence of stability in the form of a strong portfolio effect (PE) for the entire four-estuary Pinfish metapopulation, as well as the northern subcomponent of the metapopulation. Alternatively, the stability of the metapopulation across the four estuaries could have resulted from the metapopulation being well-mixed and acting as a single population. This stability could also be attributed to changes in environmental attributes causing populations to fluctuate similarly, as correlation among the separate populations of the same species can result from correlation among the variables present [65] and result in synchronization. In contrast, the low PE value for the southern component suggested that CH and TB were acting as a single population during the study period. This was further supported and explained by the strong population synchrony between the southernmost estuaries. These results suggest higher levels of stability for the full, four-estuary metapopulation as well as the northern subcomponent, compared to the southern subcomponent. The long-term stability of the Pinfish population is evident in the lack of trends in both density and biomass in this 19-year dataset, which indicates that there is currently no management concern for Pinfish stocks in the eGOM.

Intra-annual patterns of Pinfish density and biomass were synchronous across all four estuaries, despite inter-annual differences. Densities peaked earlier in the year (March-April) than biomass (April-May). This observation is consistent with recruitment of Pinfish to seagrass beds about 1 month after spawning, followed by a biomass peak as juveniles grow [64, 66, 67]. The high densities and subsequent increase in biomass in the spring months occurred when primary and secondary productivity increase in subtropical seagrass beds as water temperatures increased [30, 66]. Earlier peaks in density in CH and TB than in AB and CK could be driven by differences in larval supply, while earlier peaks in biomass could be driven by the timing of productivity increases in these subtropical latitudes being earlier than the two northern, warm-temperate systems.

Variation in growth rates could be driven by inter-annual changes in mean temperature, prey availability [68, 69], nutrient concentration, or intraspecific competition [70, 71]. Pinfish SLs increased throughout the calendar year, which is consistent with growth studies in the Gulf of Mexico and South Atlantic (Texas, Florida, and North Carolina) [22, 72]. Length at capture was consistent across all four estuaries throughout most of the year but diverged slightly in the fall months, with the largest fish in CK, smallest in AB, and intermediate fish in CH and TB. The larger SLs found in the CK region may have been due to density dependent growth, as this estuary was found to have the lowest densities, possibly reducing the strength of intraspecific competition and allowing Pinfish to grow larger. However, instantaneous growth rates for fish at CK was similar to the other estuaries, especially CH and TB. An alternative explanation is that the habitats that Pinfish used in CK left them more susceptible to the sampling gears at larger lengths than in the other systems. Instantaneous growth rates (G) varied little among estuaries, although the northernmost estuary (AB) had the lowest instantaneous growth rate. Apalachicola Bay (AB) also had the second lowest density, suggesting it may have had lower habitat quality or fewer trophic resources for Pinfish compared to the other estuaries.

The association of juvenile fishes, including Pinfish, with seagrass has been well-studied [30, 73, 74, 75, 76], and it is understood that these habitats provide both protection from predators and food for juvenile Pinfish [77, 78, 79, 80]. It is likely that higher SAV coverage aids in Pinfish larval retention within seagrass habitats and leads to higher survival compared to areas with lower SAV, which is consistent with our findings of Pinfish densities in areas with higher SAV [81]. Vegetation has been shown to influence species abundance in estuarine environments [64], which is consistent with the findings presented here of a positive effect of SAV on Pinfish presence and abundance. The strong positive effect of SAV reached a saturation level in each of the four estuaries. High density of seagrass has been shown to have a negative relationship with fish growth [82, 83] by decreasing foraging efficiency. Indeed, Spitzer et al. [84] found that in a controlled system, Pinfish foraging in higher density of seagrass had significantly lower growth than those in areas of lower seagrass coverage, and attributed this to the additional energy expended when searching for prey in more complex habitats. In an open system, it is possible that Pinfish avoid these areas in order to maximize foraging efficiency within low density seagrass habitats, leading to the observed saturating effect of SAV coverage in this study. In other systems, maximal prey and predator abundances were found within intermediate levels of habitat complexity [85].

Water temperature was also an important habitat variable related to Pinfish presence and density. Higher winter temperatures in southern estuaries may be more conducive to survival and growth of newly settled juveniles, compared to northern estuaries. The subsequent drop in density in the late summer to early fall may be explained by effects from post-settlement mortality, escapement from sampling gear by larger individuals, or perhaps offshore movement in preparation for spawning [26]. It is important to note that Pinfish abundance was significantly negatively correlated with water temperature. The increase in Pinfish density during settlement, and decrease during egress, both coincide with lower water temperatures. This relationship differs from the findings of Chacin et al. [81], which found no significant relationship between Pinfish density and water temperature in TB.

Pinfish are an important forage fish in the Gulf of Mexico that serve as a trophic link between primary and secondary production due to their herbivorous diet [16, 86, 87]. They are prey for a variety of inshore and offshore fishes [16, 31, 32, 36, 88, 89] and act as an important biologically-mediated transporter of nitrogen to offshore habitats. Indeed, Nelson et al. [16] conservatively estimated that the amount of nitrogen subsidized by the offshore migration of Pinfish from the Big Bend area alone (i.e., surrounding the CK region) was on the same order of magnitude in the eGOM as *Trichodesmium*, an important nitrogen-fixing bacteria [16]. Thus, the total amount of nutrient transfer exported via Pinfish movement to offshore food webs is likely much greater when export from the other estuaries is factored in, especially from the high-density area in the southern region of the current study. This further enhances the known importance of Pinfish to inshore-offshore trophic coupling along the WFS.

## Conclusion

Understanding broad spatial and temporal scale dynamics of forage fish populations can inform us about prey availability for higher trophic-level species of both economic and ecological importance. Pinfish meet many of the criteria for the definition of “forage fish”, and fill a similar niche for eGOM systems, especially given their important role in inshore-offshore food web coupling [16]. Overall, Pinfish abundance and biomass were the highest in areas with more SAV coverage and higher temperatures. Seagrass habitats are already influenced by a tremendous number of anthropogenic stressors including nutrient input, habitat destruction [90, 91], and increasing water temperatures from climate change which may lead to severe declines in seagrass growth and survival [92]. The potential loss of seagrass could have profound influences on many species, including Pinfish. Forage fishes have the potential to act as indicator species for the stability of higher trophic levels due to their relationships with specific habitat variables. It is therefore important to continue monitoring low trophic level forage fishes in order to better predict ecosystem-level changes.

## Supporting information

S1 Table Mean monthly numbers of Pinfish samples. Monthly mean and standard error (SE) listed for numbers of individual Pinfish sampled in each estuary.
(DOCX)
