# Supplementary material for: Population dynamics of Pinfish in the eastern Gulf of Mexico (1998-2016)
Source: PLoS One. 2019 Aug 22;14(8):e0221131. doi: 10.1371/journal.pone.0221131 (PMC6705858; doi:10.1371/journal.pone.0221131)
Supplement: S1 Table — Monthly mean and standard error (SE) listed for numbers of individual Pinfish sampled in each estuary. (DOCX) [file pone.0221131.s001.docx]

| **Bay** | **Mean** | **SE** | **Min** | **Max** |
| --- | --- | --- | --- | --- |
| AP | 9.94 | 0.17 | 1 | 12 |
| CH | 15.47 | 0.15 | 11 | 18 |
| CK | 12.00 | 4.39e-03 | 11 | 12 |
| TB | 20 | 0 | 20 | 20 |

**S1 Table. Mean monthly numbers of seine hauls.** Monthly mean and standard error (SE) listed for number of seine hauls in each estuary.
